# Prognostic Value, Clinicopathologic Features and Diagnostic Accuracy of Interleukin-8 in Colorectal Cancer: A Meta-Analysis

**DOI:** 10.1371/journal.pone.0123484

**Published:** 2015-04-09

**Authors:** Wenjie Xia, Wuzhen Chen, Zhigang Zhang, Dang Wu, Pin Wu, Zhigang Chen, Chao Li, Jian Huang

**Affiliations:** 1 Department of Oncology, Second Affiliated Hospital, Zhejiang University School of Medicine, Hangzhou, China; 2 Cancer Institute, Key Laboratory of Cancer Prevention&Intervention, National Ministry of Education, Provincial Key Laboratory of Molecular Biology in Medical Sciences, Zhejiang University School of Medicine, Hangzhou, China; Heinrich-Heine-University and University Hospital Duesseldorf, GERMANY

## Abstract

**Background:**

The prognostic value and diagnostic accuracy of Interleukin-8 (IL-8) in colorectal cancer have been assessed with several studies, but the conclusions were inconclusive. Thus we performed a meta-analysis to evaluate the impact of IL-8 expression on colorectal cancer prognosis, clinicopathologic features and diagnostic accuracy.

**Methods:**

Comprehensive search strategies were used to search relevant literature in the PubMed, EBSCO and the ISI Web of Science databases. The correlation between IL-8 expression and prognosis, clinicopathologic features and diagnostic accuracy was analyzed.

**Results:**

A total of 18 articles met the inclusion criteria, including 1509 patients for clinicopathologic features or prognosis evaluation and 725 participants for diagnostic evaluation. The results suggested that overexpression of IL-8 was significantly associated with poor prognosis in colorectal cancer (HR = 1.54, 95%CI 1.03–2.32), especially in Union for International Cancer Control (UICC) stage IV patients (HR = 2.28, 95%CI 1.60–3.25). With further subgroup analysis, we found that high IL-8 level in serum was significantly correlated with poor prognosis (HR = 2.13, 95%CI 1.49–3.05). In addition, significant correlations were observed between high IL-8 expression and advanced stage (OR = 3.01, 95%CI 1.98–4.56), lymphatic metastasis (OR = 2.24, 95%CI 1.39–3.63), and liver metastasis (OR = 3.47, 95%CI 1.74–6.89). Moreover, IL-8 had high diagnostic accuracy, with pooled sensitivity 0.70(95%CI 0.66–0.74), specificity 0.91(95%CI 0.86–0.94), positive likelihood ratio (LR) 7.00(95%CI 2.48–19.73), negative LR 0.24(95%CI 0.09–0.64), diagnostic OR 24.00(95%CI 5.52–104.38).

**Conclusions:**

This study showed that IL-8 could be a potential indicator for detecting colorectal cancer and predicting prognosis. In addition, high IL-8 level was significantly correlated with advanced stage, lymphatic metastasis, liver metastasis.

## Introduction

Colorectal cancer(CRC) is the third most frequent cancer in the world, and is one of the leading causes of cancer related death[1]. Despite the satisfied 5-year survival rate of early stage of Union for International Cancer Control(UICC), the prognosis of advanced stage is very poor, especially for stage IV(5-year overall survival <5%)[2]. Thus it is essential to find efficient indicators for detecting cancer early and assessing disease status properly.

Chemotactic cytokines play an important role in angiogenesis and drawing immune cells. However, their impact on tumor formation remains incompletely understood[3]. Recently, growing evidence indicates that chemotactic cytokines may be associated with cancers, and could be potential indicators for detecting cancers and predicting prognosis beyond conventional clinicopathologic indicators[4]. Interleukin-8(IL-8), also called chemokine (C-X-C motif) ligand 8(CXCL8), is the first inflammatory chemokine discovered in 1987[5]. As a member of the CXC cytokine family, IL-8 could induce angiogenesis and chemotaxis of many kinds of cells, such as neutrophils, macrophages, endothelial cells, and cancer cells[6]. During infiammatory process in colorectal cancer, IL-8 is induced by NF-κB pathway, which may result in an increased number of tumor vessels[7]. However, there is insufficient evidence to confirm the prognostic value and diagnostic accuracy of IL-8 in colorectal cancer. Some studies revealed that IL-8 had a higher expression in melanoma, glioblastoma, non-small-cell lung carcinoma, and colorectal cancer, and through binding to G protein-coupled receptor CXCR1 and CXCR2, it was closely linked with angiogenesis, tumor growth, metastasis and survival[8–11]. But some other studies suggested IL-8 was not linked with tumor progression and worse prognosis in colorectal cancer[3], and CXCL8-positivity in the tumor infiltrate might be associated with a statistically significant reduction in the risk of disease recurrence[12].

To address this issue, we conducted a meta-analysis of all eligible studies to evaluate the impact of IL-8 expression on CRC diagnostic accuracy, clinicopathologic features and prognosis, and to find if IL-8 could be a potential efficient indicator for detecting colorectal cancer or predicting prognosis.

## Materials and Methods

### Data sources and selection criteria

We searched literature from PubMed, EBSCO and the ISI Web of Science databases with the terms: ‘‘IL-8”,” interleukin-8”, ‘‘colorectal neoplasm”, ‘‘colorectal cancer”, “CRC” with all possible combinations. And reference lists of review articles, bibliographies, or some other relevant studies were also searched manually for finding the additional eligible studies. The last search was performed on August 17, 2014.

The inclusion criteria for selecting articles in the meta analysis were as follows: (1) the included studies must evaluate IL-8 expression in the tissues or serum of CRC patients; (2)studies must evaluate the relationships between IL-8 and CRC diagnostic accuracy, clinicopathologic features or prognosis; (3)studies must give us sufficient data to estimate hazard ratio(HR) for overall survival(OS), odds ratio(OR) for clinicopathologic features, or true positive(TP), false positive(FP), false negative(FN), true negative(TN) for diagnostic evaluation; (4)articles written in English.

Conference abstracts, reviews, letters and case reports were excluded due to the insufficiency to properly assess the relevant study data and characteristics. And if several different studies contained the same patient population, we just included the most complete study to avoid duplication.

### Data extraction and quality assessment

Two authors (Dang Wu and Wenjie Xia) reviewed and extracted data from each eligible study independently. Controversial problems were resolved by the third investigator Wuzhen Chen. The following data were collected for each eligible study: the first author’s name, year of publication, number of patients, patients characteristics, country of origin, detection methods, cancer characteristics, cutoff value, number of TP, FP, FN, TN, survival data and follow-up. Relevant data were summarized and described in table format.

We used Newcastle–Ottawa quality assessment scale (NOS) to evaluate the quality of each included cohort study and Quality Assessment of Diagnostic Accuracy Studies (QUADAS) to evaluate the quality of each included diagnostic accuracy study[13, 14]. The NOS assessed each study with eight items in three aspects: selection, comparability, and outcome. Each item could gain 1 score except for the item related to comparability which allowed 2 scores. The studies with 6 scores or more were classified as high quality studies. And similarly, the QUADAS assessed each study with 14 items, and the studies with 9 scores or more were classified as high quality studies. Two researchers independently assessed studied and gave scores, and disagreements were solved by discussion. Finally a consensus value for each item was achieved.

### Statistical analysis

For the pooled analysis of the effect of IL-8 expression on CRC patients survival, hazard ratio(HR) and its 95% CI were extracted from each eligible study: If HR data were described directly, we just took them; otherwise, they were estimated from available data in the articles with the methods described by Parmar and Jayne[15, 16]. HR data were combined for evaluating the association between IL-8 expression and overall survival. Odds ratio(OR) and its 95% CI were combined for assessing the relation between IL-8 expression and CRC clinicopathological features, such as tumor stage, lymphatic metastasis, and liver metastasis. And we extracted TP, FP, FN, TN for evaluating diagnostic accuracy of IL-8. Analysis was conducted with STATA (v12.0; Stata Corp LP, TX, USA) and Meta-Disc 1.4 for Windows (XI Cochrane Colloquium, Barcelona, Spain). Heterogeneity was evaluated with Chi-square based Q statistical test and the I2 statistic[17, 18]. When heterogeneity was not remarkable (I2 values < 50%), a fixed-effects model was used[19]. Otherwise, we used the random-effects model to pool data[20]. Publication bias was assessed by Begg’s test(for publication bias in clinicopathologic features or prognosis evaluation) and Deeks’ funnel plot asymmetry test(for publication bias in diagnostic tests), if p<0.05, publication bias was indicated[21].

## Results

### Identification of relevant studies

**Fig 1:**
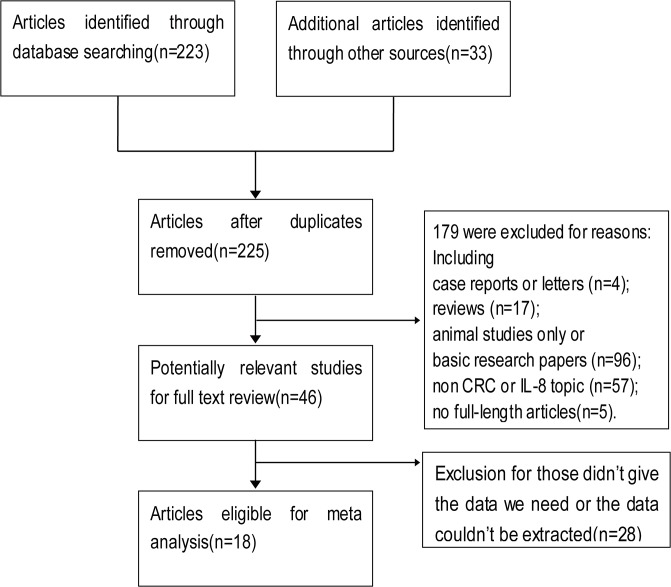
Flow diagram of study selection procedure.

As shown in **Fig 1**, 225 published articles in total were identified with the search strategy described above. After exclusion for reasons, such as reviews, basic research papers, non CRC or IL-8 topic, totally 46 articles were included for full text review, and in the review, 28 articles were excluded for reasons(list of full-text excluded articles and reasons for exclusion could be seen in **S1 Appendix**). Finally, a total of 18 eligible studies were included in the final meta-analysis[3, 8, 12, 22–36]. Of these 18 publications, 15 articles evaluated the association between IL-8 expression and clinicopathologic features or prognosis of colorectal cancer, and 5 were for diagnostic evaluation. The clinical features of eligible studies were listed in **Table 1** and **Table 2**. Totally, 1509 patients were included for clinicopathologic features or prognosis evaluation, with a median of 101(from 22 to 228) per study; and 725 participants were included for diagnostic evaluation, with a median of 145(from 71 to 218) per study. Within 15 studies for clinicopathologic features or prognosis evaluation, 6 were from Asia, 3 were from North America, 5 were from Europe, and 1 was from Oceania. 14 of these studies got 6 scores or more in quality assessment, and were classified as high quality studies. While for 5 diagnostic evaluation studies, 2 were from Asia, 3 were from Europe, and all studies were classified as high quality studies.

**Table 1:** Basic characteristics of the included studies.

**First author**	**Year**	**Country**	**Participants**	**Methods**	**Cutoff value**	**TP**	**FP**	**FN**	**TN**	**QUADAS score**
George Sgourakis	2014	Germany	71	ELISA	8.83 pg/ml	48	3	8	12	12
Zhang Pengjun	2013	China	218	Magnetic Beads	44.26 pg/ml	129	2	20	67	12
Stefanie Bunger	2012	Germany	133	biochips	39.5 pg/ml	18	5	63	47	10
ZHI YAN	2012	China	96	PCR	NA	45	11	3	37	10
J. Kaminska	2005	Poland	207	ELISA	17.71 pg/ml	106	1	51	49	11

NA, not available; ELISA, enzyme linked immunosorbent assay; PCR, polymerase chain reaction; TP, true positive; FP, false positive; FN, false negative; TN, true negative; QUADAS, Quality Assessment of Diagnostic Accuracy Studies.

**Table 2:** Basic characteristics of the included studies.

**First author**	**Year**	**Country**	**Patients**	**Median Age**	**Methods**	**Follow up**	**Cutoff value**	**HR estimation**	**NOS score**
Xian-Shuo Cheng	2014	China	213	62	IHC	61 months	Score>4	Survival curves for OS	8
Zhi-Yuan Chen	2014	China	176	58	Magnetic Beads	19.6 months	9.58 pg/mL	HR for OS	6
Maressa A. Bruhn	2014	Australia	155	NA	MPA	30.2 months	1.21 pg/mL	HR for OS	5
Trevor D Hamilton	2014	Canada	70	61	MPA	20 months	NA	HR for OS	7
Yingmiao Liu	2013	America	38	53.5	ELISA	40 months	NA	HR for OS	6
Shimazaki J	2013	Japan	46	70.3	ELISA	NA	8.0 pg/ml	HR for OS	6
Stefanie Bunger*	2012	Germany	164	69.6	biochips	NA	39.5pg/ml	NA	7
T Kantola	2012	Finland	115	67.9	Cytokine Panel	NA	NA	NA	7
ZHI YAN*	2012	China	48	58.6	PCR	80 months	NA	HR for OS	7
Oladipo	2011	The UK	228	64	IHC	68 months	≥2 cores positive	HR for OS	8
Dietrich Doll	2010	Germany	90	64	PCR	76 months	2.41^a^	Survival curves for OS	8
Charles Bailey	2007	The UK	22	NA	RPA	NA	Grade>2	NA	6
Daniel Vallbohmer	2005	America	33	64	PCR	7.1 months	4.81*10^-3^b^	HR for OS	6
H. Terada	2005	Japan	87	NA	ELISA	60 months	3,000 pg/mg	HR for OS	7
TAKASHI UEDA	1994	Japan	24	NA	ELISA	NA	50%	NA	6

IHC, immunohistochemistry; MPA, multiplex protein assay; RPA, ribonuclease protection assay; HR, hazard ratio; OS, overall survival; NOS, Newcastle–Ottawa quality assessment scale

a Relative to hypoxanthine-phosphoribosyl-transferase

b Relative to β-actin

*Date from the same study mentioned before.

### Impact of IL-8 expression on overall survival of colorectal cancer

**Fig 2:**
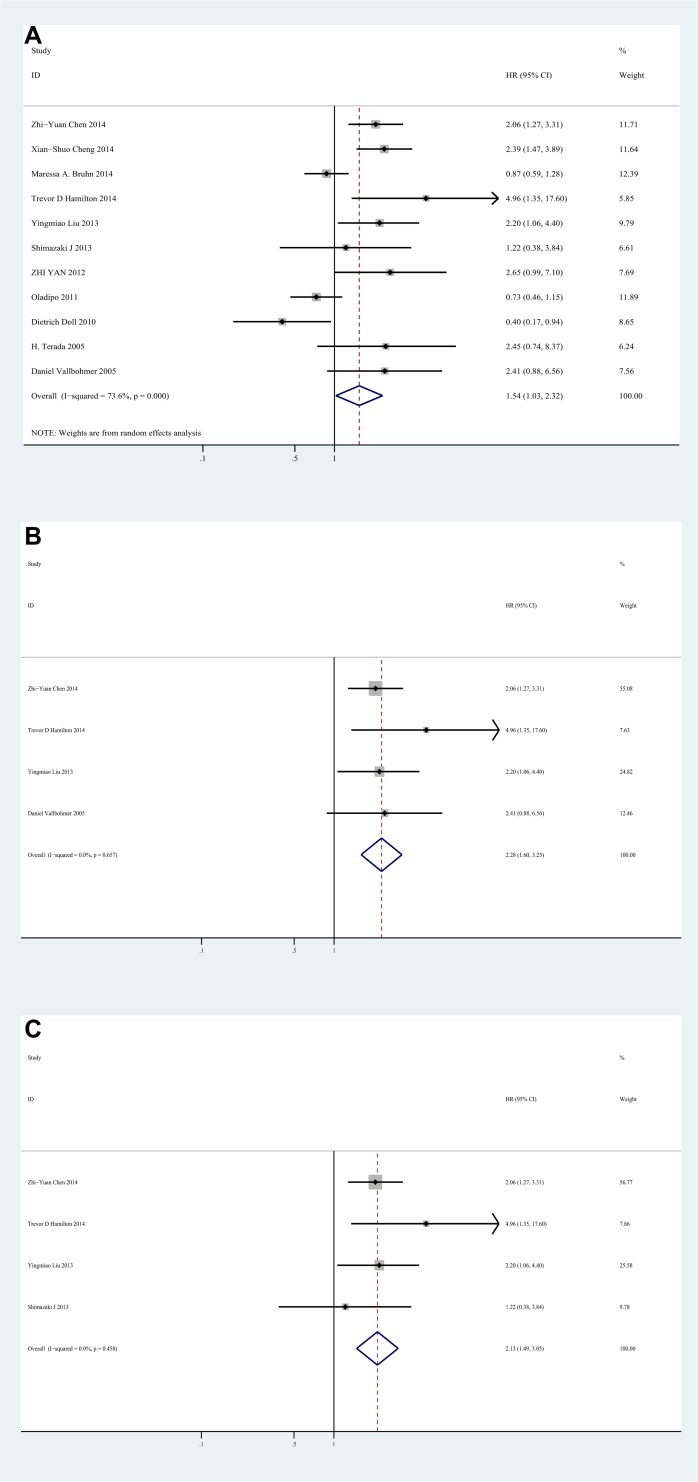
Forest plot of Hazard ratio (HR) for the association between Interleukin-8 and overall survival(OS). **A.** HRs with corresponding 95% CIs of Interleukin-8 expression with OS. **B.** HRs with corresponding 95% CIs of Interleukin-8 expression in stage IV colorectal cancer patients with OS. **C.** Subgroup analysis of HRs with corresponding 95% CIs of serum source Interleukin-8 expression with OS. HR>1 implied worse survival for the group.

11 studies evaluated the impact of IL-8 expression on overall survival of CRC with HRs. The pooled HR was 1.54(95%CI 1.03–2.32, I2 = 73.6%, random effect) **(Fig 2A)**. It suggested that high IL-8 level significantly increased the mortality risk when compared with low IL-8 level for CRC patients. And in high quality studies, the pooled HR of UICC stage IV subgroup was 2.28(95%CI 1.60–3.25, I2 = 0%, fixed effect) **(Fig 2B)**, indicating that high IL-8 expression was significantly correlated with poor prognosis for stage IV CRC patients. Additionally, in subgroup analysis based on the source of IL-8, poor OS in CRC was correlated with the overexpression of IL-8 in serum, with the pooled HR of 2.13(95%CI 1.49–3.05, I2 = 0%) **(Fig 2C)**.

We also performed subgroup analysis by study location, study size, median age and follow-up, it indicated a significant correlation between high IL-8 expression and poor prognosis in Asian studies, with the pooled HR of 2.17(95%CI 1.61–2.94, I2 = 0%), while not for studies from other areas. And high IL-8 expression was also significantly correlated with poor prognosis in median age≤60 subgroup and follow-up≤60 months subgroup, with the pooled HR of 2.17(95%CI 1.50–3.13, I2 = 0%) and 1.93(95%CI 1.15–3.23, I2 = 67.1%) respectively **(S1 Table)**.

### Correlation between IL-8 and clinicopathological parameters

**Fig 3:**
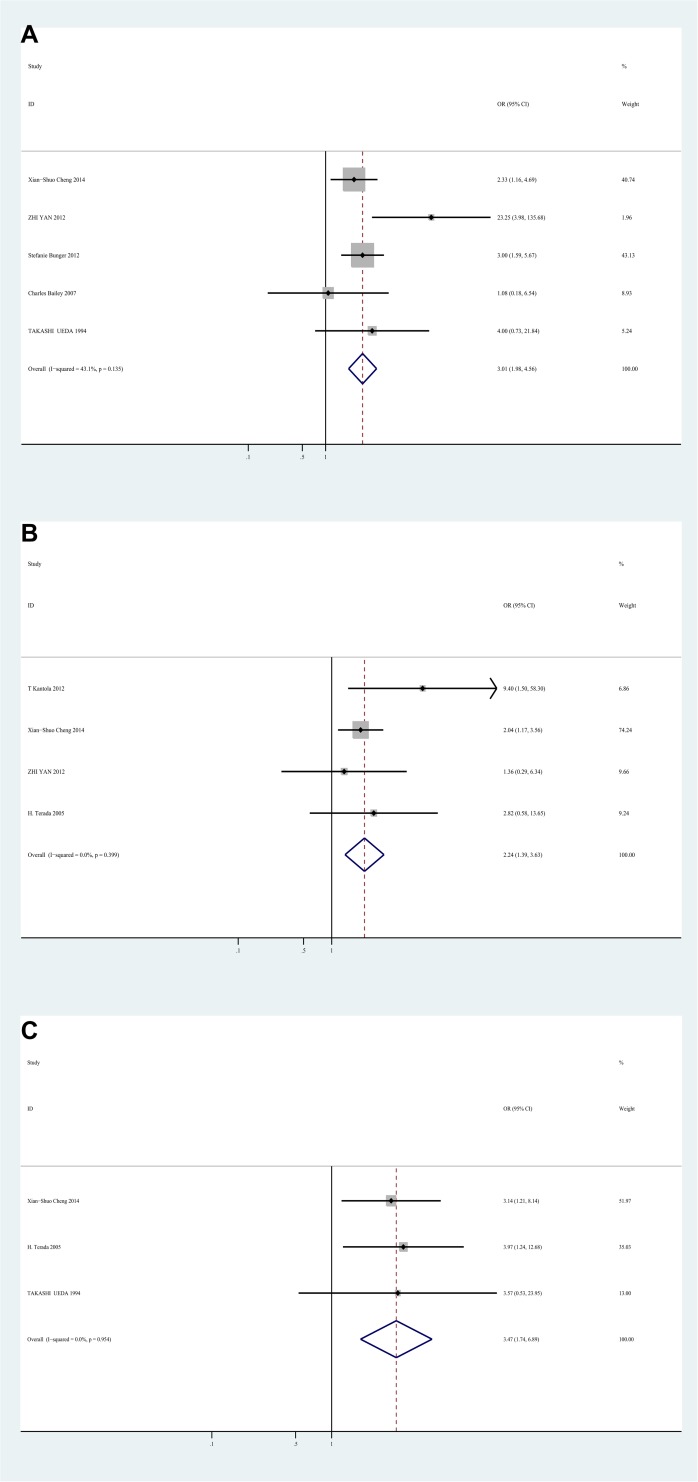
Forest plot of Odds ratio (OR) for the association between Interleukin-8 and clinicopathological features. **A.** ORs with corresponding 95% CIs of Interleukin-8 expression with UICC stage. **B.** ORs with corresponding 95% CIs of Interleukin-8 expression with lymphatic metastasis. **C.** ORs with corresponding 95% CIs of Interleukin-8 expression with liver metastasis. OR>1 implied significant correlations between IL-8 and advanced stage, lymphatic metastasis, and liver metastasis.

The meta-analysis also evaluated the correlation between IL-8 expression and clinicopathological characteristics of CRC. High level of IL-8 was significantly correlated with advanced stage (UICC stage), with the OR of 3.01 (95%CI 1.98–4.56, I2 = 43.1%, fixed effect) **(Fig 3A)**. In addition, high IL-8 expression was significantly associated with lymphatic metastasis (OR = 2.24, 95%CI 1.39–3.63, I2 = 0%, fixed effect) **(Fig 3B)**, and liver metastasis (OR = 3.47, 95%CI 1.74–6.89, I2 = 0%, fixed effect) **(Fig 3C)**, indicating that IL-8 might be a potential indicator for metastasis of colorectal cancer. However, there was no significant correlation between IL-8 expression and differentiation (poor vs well; OR = 1.04, 95%CI 0.58–1.87, I2 = 0%, fixed effect), gender (male vs female; OR = 1.31, 95%CI 0.90–1.90, I2 = 23%, fixed effect), age (older vs younger; OR = 1.07, 95%CI 0.69–1.64, I2 = 0%, fixed effect), and site (rectum vs colon; OR = 1.35, 95%CI 0.88–2.06, I2 = 35%, fixed effect)**(S2 Table)**.

### Diagnostic accuracy of IL-8 for diagnosing colorectal cancer

**Fig 4:**
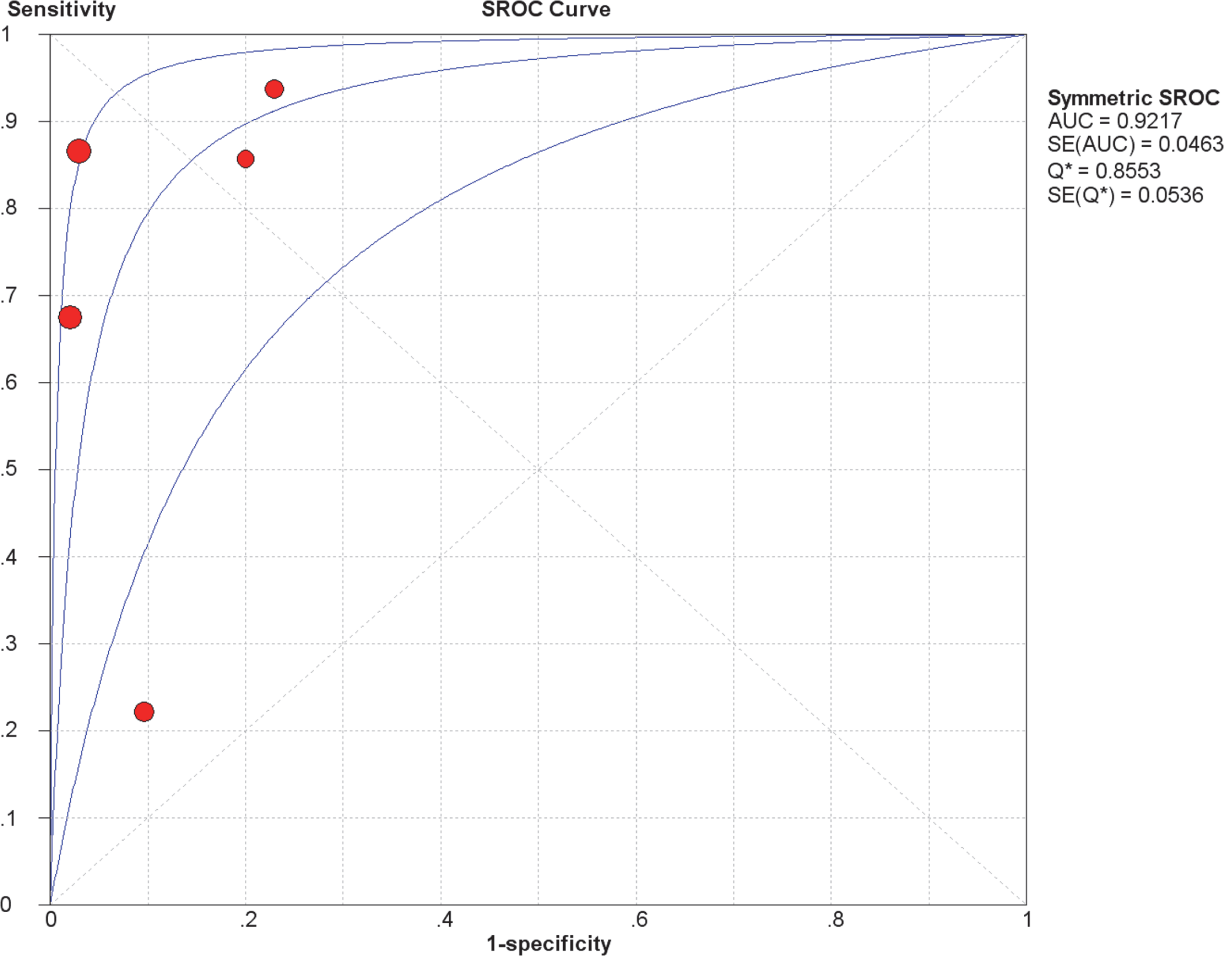
The Summary receiver operating characteristic (SROC) curve of Interleukin-8 for detecting colorectal cancer. The AUC is 0.92.

5 studies were included for evaluating the diagnostic accuracy of IL-8. The Spearman test showed there was no threshold effect (Spearman correlation coefficient = 0.500, p = 0.391). The pooled sensitivity was 0.70(95%CI 0.66–0.74), specificity was 0.91(95%CI 0.86–0.94), positive likelihood ratio (LR) was 7.00(95%CI 2.48–19.73), negative LR was 0.24(95%CI 0.09–0.64), diagnostic OR was 34.93(95%CI 6.40–190.70)**(Table 3)**. Summary receiver operating characteristic curve (SROC curve) was shown in **Fig 4**, with the AUC of 0.92. Meta regression was used for finding the source of heterogeneity, and quality (with QUADAS scores), design (with or without blind and random) and methods (such as source of IL-8, detecting methods) did not reach statistical significance. And with sensitivity analysis, we found if we deleted an article (Stefanie Bunger), the heterogeneity could significantly drop down **(Table 4)**, thus it might be the source of heterogeneity. The SROC curve and the area under the curve indicated that IL-8 represented a high level of diagnostic accuracy.

**Table 3:** Diagnostic parameters of IL-8 for detecting colorectal cancer.

**Parameters**	**Pooled ratio**	**Low value of 95%CI**	**High value of 95%CI**	**I square**	**Model used**
Sensitivity	0.70	0.66	0.74	96.8%	Random effect model
Specificity	0.91	0.86	0.94	78.2%	Random effect model
Positive LR	7.00	2.48	19.73	80.7%	Random effect model
Negative LR	0.24	0.09	0.64	97.9%	Random effect model
Diagnostic OR	34.93	6.40	190.70	85.9%	Random effect model

LR: likelihood ratio; OR: odds ratio; CI: confidence interval.

**Table 4:** Sensitivity analysis of IL-8 for diagnostic evaluation.

**Study omitted**	**Pooled diagnostic OR**	**Low value of 95%CI**	**High value of 95%CI**	**I square**
George Sgourakis	39.15	4.40	348.22	89.4%
Zhang Pengjun	21.76	3.92	120.70	82.8%
Stefanie Bunger	68.71	25.72	183.54	38.0%
ZHI YAN	32.41	3.65	288.05	88.9%
J. Kaminska	27.82	4.00	193.64	88.3%

### Publication bias

Begg’s test and Deeks’ funnel plot asymmetry test indicated there was no proof of obvious publication bias after evaluating the funnel plot for the studies included in the meta-analysis (**S1** and **S2 Figs**).

## Discussion

Tumor growth and metastasis are affected by a group of soluble mediators called chemokines, and there is growing evidence that by regulating balance between angiogenic and anti-angiogenic, chemokines could affect the outgrowth or shrinkage of tumors[3]. IL-8 is a member of CXC chemokine family, and has been reported to be associated with various cancers, such as gastric cancer, colorectal cancer[35]. In addition, IL-8 was reported to be able to activate the classical MAPK signaling cascade, with downstream phosphorylation of Erk1/2 in neutrophils and cancer cells. And by IL-8, activation of MAPK signaling is consistent with the improvement of proliferation and survival for various types of cells[37]. What’s more, stimulated with IL-8, focal adhesion kinase (FAK) and Src-kinases are also activated in cancer cells, which are also consistent with the improvement of proliferation, survival, and chemoresistance[37, 38]. Many studies were performed to evaluate the correlation between IL-8 and colorectal cancer, but the accurate relation hasn’t been confirmed sufficiently.

To our knowledge, this meta-analysis is the first study to systematically evaluate the impact of IL-8 expression on prognostic factors, and clinicopathological features in colorectal cancer.

In our study, 15 studies were included to assess the association between IL-8 expression and clinicopathologic features or prognosis of colorectal cancer, and 5 were for diagnostic evaluation. Finally, we found high level of IL-8 indicated poor prognosis in colorectal cancer, especially for UICC stage IV CRC patients. It suggested that IL-8 might be an efficient predictor of poor OS, especially for mCRC patients. And high IL-8 expression was significantly associated with poor overall survival in Asian countries, while not in other countries. It indicated that IL-8 overexpression seemed to be correlated with unfavorable prognosis and tumor progress in Asian colorectal cancer patients. In addition, Overexpression of IL-8 was significantly correlated with poor prognosis in median age≤60 subgroup, suggesting that IL-8 might indicate unfavorable prognosis in younger CRC patients. What’s more, we found that high IL-8 level in serum was significantly correlated with poor prognosis, indicating it might be a potential convenient and efficient method for predicting prognosis of colorectal cancer and have a good clinical application value. The liver is the most common site of colorectal cancer metastases, affecting approximately 25–35% of all[39]. Thus we choose liver metastasis as a representative for evaluating the correlation between IL-8 expression and colorectal cancer metastases. And significant correlations were observed between high IL-8 expression and advanced stage, lymphatic metastasis and liver metastasis, indicating IL-8 might be a potential indicator for tumor progress and metastasis. We further found IL-8 had high diagnostic accuracy, suggesting that it might be a potential indicator for both detecting colorectal cancer early and predicting prognosis properly.

Hu et al. (2012) preformed a meta-analysis which indicated there was no obvious association between Interleukin-8-251 T>A polymorphism and colorectal cancer risk[40]. However, we think this is not conflict with our findings that IL-8 might be a potential indicator for both detecting colorectal cancer early and predicting prognosis properly. Polymorphism of IL-8 is thought to be able to influence production and expression of IL-8, while it isn’t the unique cause to affect the expression of IL-8. IL-8 could also be affected by various reasons, and in the context of a tumor, IL-8 could be induced, and is known to participate in cancer progression by promoting the angiogenic response, the recruitment of neutrophils to the site of the tumor, and the proliferation, survival and migration of tumor cells[41, 42]. Furthermore, for each person the polymorphism will not vary, while the expression of IL-8 could change a lot according to the condition. Thus the two conclusions don’t conflict.

Jin et al. (2014) studied the diagnostic value of IL-8 in colorectal cancer and thought IL-8 was a promising biomarker for CRC detection[43]. However, by including the study published by Kantola et al. (2012) [24], they extracted data from a ROC curve that was established for multiple serum cytokines, not for IL-8 only. Thus we amended and updated the included articles, and the results might be more suitable for assessing the diagnostic value of IL-8 in colorectal cancer.

Some limitations exist in the present meta-analysis. First, the number of included studies was relatively small, mainly for the reason that IL-8 expression was a continuous variable, thus some other studies just gave the mean value of IL-8 other than evaluated it with high or low, and we were unable to assess it. Second, the methods for detecting IL-8 expression and the cutoff values were various in the included studies, which could cause heterogeneity among the studies. And we could not perform subgroup analysis to explore this impact for lack of sufficient data. Third, as there was no acknowledged cut off point of IL-8, we just used the cutoff point the original articles gave for diagnostic evaluation. And this cutoff point usually was closest to the point with both maximum sensitivity and specificity, thus it would overestimated the diagnostic accuracy of IL-8. But all included articles except for one indicated AUC of IL-8>0.8, thus the bias wouldn’t be very large. Although the AUC of 0.92 demonstrated a high level of diagnostic accuracy, other data were suboptimal. This was mainly caused by a large heterogeneity and relatively small numbers of study patients. To better evaluate the diagnostic accuracy of IL-8, we need more studies with standard detecting methods and cutoff value.

In summary, high IL-8 expression was significantly correlated with poor prognosis in colorectal cancer, especially for stage IV. And significant correlations were identified between high IL-8 expression and poor prognosis in Asian, serum, age≤60 and follow-up≤60 months subgroups. Additionally, overexpression of IL-8 was significantly correlated with tumor stage, lymphatic metastasis and liver metastasis. What’s more, IL-8 had high diagnostic accuracy. Thus IL-8 might be a potential efficient indicator for both detecting cancers and predicting prognosis.

## Supporting Information

S1 PRISMA Checklist PRISMA Checklist. (DOC)

S1 Fig Begg’s funnel plot for detecting publication bias. **A.** Begg’s funnel plot showed no significant publication bias for studies assessing Interleukin-8 expression and OS in colorectal cancer patients. **B.** Begg’s funnel plot showed no significant publication bias for studies assessing Interleukin-8 expression and UICC stage in colorectal cancer patients. **C.** Begg’s funnel plot showed no significant publication bias for studies assessing Interleukin-8 expression and lymphatic metastasis in colorectal cancer patients. **D.** Begg’s funnel plot showed no significant publication bias for studies assessing Interleukin-8 expression and liver metastasis in colorectal cancer patients.
(TIF)

S2 Fig Deeks’ funnel plot asymmetry test for detecting publication bias. Deeks’ funnel plot asymmetry test showed no significant publication bias in diagnostic tests.
(TIF)

S1 Table Stratified analysis of pooled hazard ratios for colorectal cancer patients with IL-8. (DOCX)

S2 Table Correlation between IL-8 expression and clinicopathological features. (DOCX)

S1 Appendix List of full-text excluded articles and reasons for exclusion. (DOCX)
