# Supplementary figures and images for: Prognostic Value, Clinicopathologic Features and Diagnostic Accuracy of Interleukin-8 in Colorectal Cancer: A Meta-Analysis
Source: PLoS One. 2015 Apr 9;10(4):e0123484. doi: 10.1371/journal.pone.0123484 (PMC4391830; doi:10.1371/journal.pone.0123484)

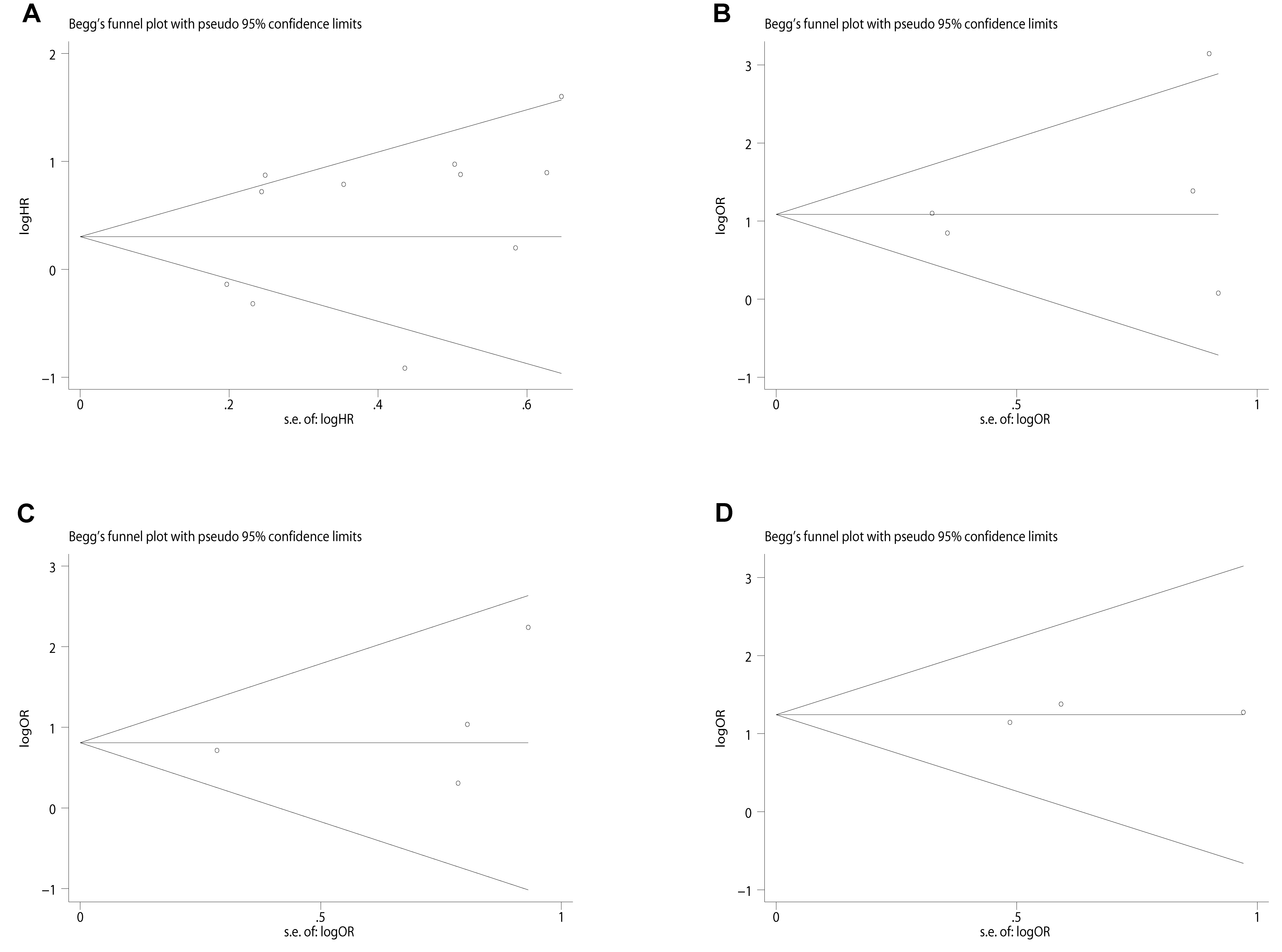

Supplement: S1 Fig — A. Begg’s funnel plot showed no significant publication bias for studies assessing Interleukin-8 expression and OS in colorectal cancer patients. B. Begg’s funnel plot showed no significant publication bias for studies assessing Interleukin-8 expression and UICC stage in colorectal cancer patients. C. Begg’s funnel plot showed no significant publication bias for studies assessing Interleukin-8 expression and lymphatic metastasis in colorectal cancer patients. D. Begg’s funnel plot showed no significant publication bias for studies assessing Interleukin-8 expression and liver metastasis in colorectal cancer patients. (TIF) [file pone.0123484.s002.tif]

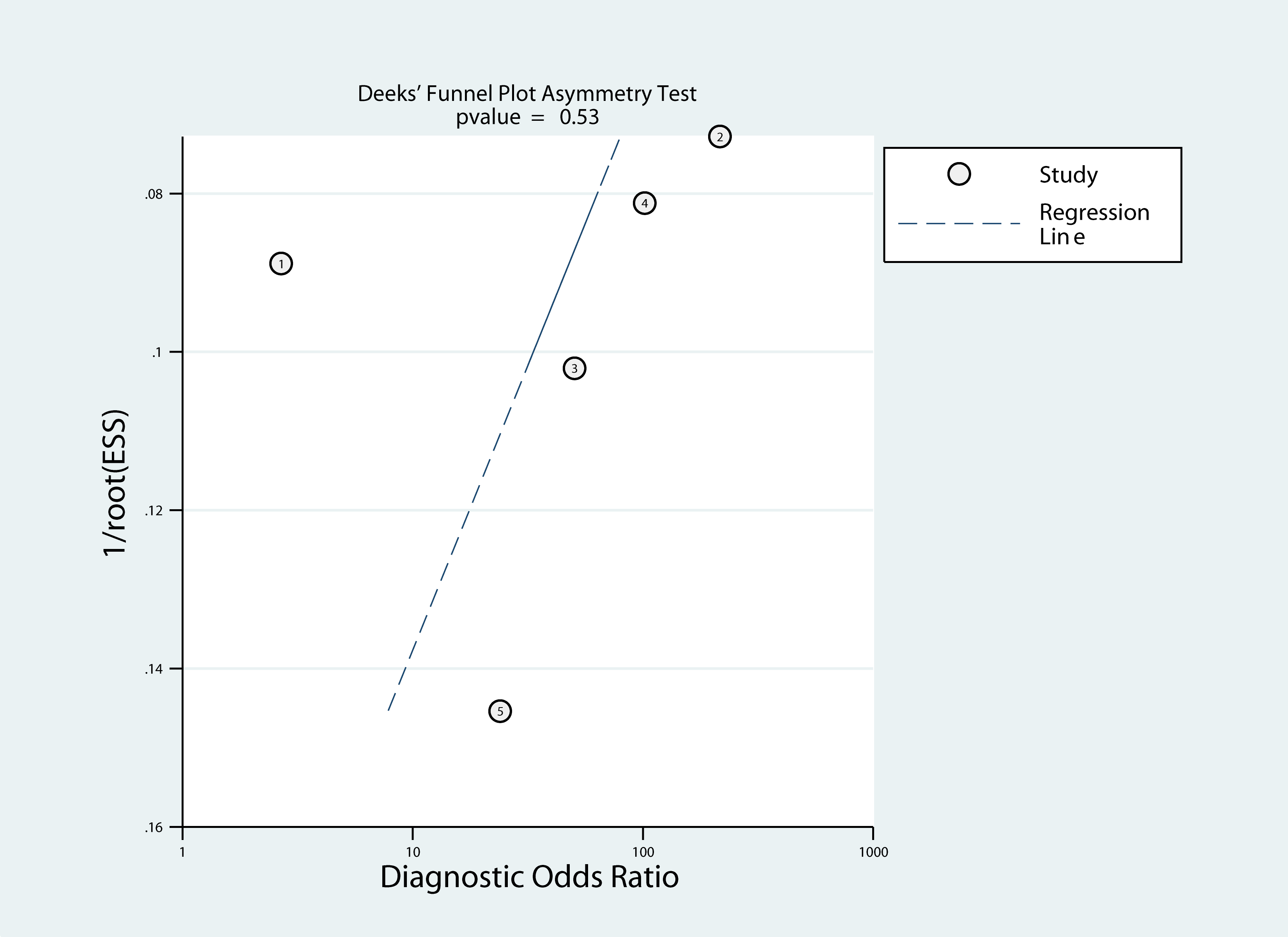

Supplement: S2 Fig — Deeks’ funnel plot asymmetry test showed no significant publication bias in diagnostic tests. (TIF) [file pone.0123484.s003.tif]
