# Supplementary material for: Prognostic Value, Clinicopathologic Features and Diagnostic Accuracy of Interleukin-8 in Colorectal Cancer: A Meta-Analysis
Source: PLoS One. 2015 Apr 9;10(4):e0123484. doi: 10.1371/journal.pone.0123484 (PMC4391830; doi:10.1371/journal.pone.0123484)
Supplement: S1 Table — (DOCX) [file pone.0123484.s004.docx]

**Table S1. Stratified analysis of pooled hazard ratios for colorectal cancer patients with IL-8.**

| **Stratified analysis** | **Number of studies** | **Number of patients** | **Pooled HR(95%CI)** | **I square** | **Model used** |
| --- | --- | --- | --- | --- | --- |
| Study size |  |  |  |  |  |
| >100 | 4 | 772 | 1.32(0.74-2.34) | 84.8% | Random effect model |
| ≤100 | 7 | 412 | 1.80(0.97-3.33) | 62.7% | Random effect model |
| Median Age |  |  |  |  |  |
| >60 | 6 | 680 | 1.39(0.69-2.79) | 80.1% | Random effect model |
| ≤60 | 3 | 262 | 2.17(1.50-3.13) | 0% | Fixed effect model |
| Follow up(month) |  |  |  |  |  |
| >60 | 4 | 579 | 1.16(0.50-2.71) | 85.8% | Random effect model |
| ≤60 | 6 | 559 | 1.93(1.15-3.23) | 67.1% | Random effect model |
| Study location |  |  |  |  |  |
| Asian | 5 | 570 | 2.17(1.61-2.94) | 0% | Fixed effect model |
| Non-Asian | 6 | 614 | 1.21(0.69-2.12) | 75.5% | Random effect model |
