# Supplementary material for: Prognostic Value, Clinicopathologic Features and Diagnostic Accuracy of Interleukin-8 in Colorectal Cancer: A Meta-Analysis
Source: PLoS One. 2015 Apr 9;10(4):e0123484. doi: 10.1371/journal.pone.0123484 (PMC4391830; doi:10.1371/journal.pone.0123484)
Supplement: S2 Table — (DOCX) [file pone.0123484.s005.docx]

**Table S2.** **Correlation between IL-8 expression and clinicopathological features.**

| **Clinical features** | **Pooled OR** | **Low value of 95%CI** | **High value of 95%CI** | **I square** | **Model used** |
| --- | --- | --- | --- | --- | --- |
| Differentiation (poor vs well) | 1.04 | 0.58 | 1.87 | 0% | Fixed effect model |
| Gender (male vs female) | 1.31 | 0.90 | 1.90 | 23% | Fixed effect model |
| Age (older vs younger) | 1.07 | 0.69 | 1.64 | 0% | Fixed effect model |
| Site (rectum vs colon) | 1.35 | 0.88 | 2.06 | 35% | Fixed effect model |
