# Supplementary material for: Prognostic Value, Clinicopathologic Features and Diagnostic Accuracy of Interleukin-8 in Colorectal Cancer: A Meta-Analysis
Source: PLoS One. 2015 Apr 9;10(4):e0123484. doi: 10.1371/journal.pone.0123484 (PMC4391830; doi:10.1371/journal.pone.0123484)
Supplement: S1 Appendix — (DOCX) [file pone.0123484.s006.docx]

**Appendix S1. List of full-text excluded articles and reasons for exclusion.**

| **Excluded articles[**[**1-28**](#_ENREF_1)**]** | **Reasons for exclusion** |
| --- | --- |
| 1. Abajo A, Boni V, Lopez I, Gonzalez-Huarriz M, Bitarte N, et al. (2012) Identification of predictive circulating biomarkers of bevacizumab-containing regimen efficacy in pre-treated metastatic colorectal cancer patients. Br J Cancer 107: 287-290.    2. Giessen C, Nagel D, Glas M, Spelsberg F, Lau-Werner U, et al. (2014) Evaluation of preoperative serum markers for individual patient prognosis in stage I-III rectal cancer. Tumour Biol 35: 10237-10248.    3. Kopetz S, Hoff PM, Morris JS, Wolff RA, Eng C, et al. (2010) Phase II trial of infusional fluorouracil, irinotecan, and bevacizumab for metastatic colorectal cancer: efficacy and circulating angiogenic biomarkers associated with therapeutic resistance. J Clin Oncol 28: 453-459.    4. Ferri M, Rossi Del Monte S, Salerno G, Bocchetti T, Angeletti S, et al. (2013) Recovery of immunological homeostasis positively correlates both with early stages of right-colorectal cancer and laparoscopic surgery. PLoS One 8: e74455.    5. Dimberg J, Strom K, Lofgren S, Zar N, Lindh M, et al. (2012) DNA promoter methylation status and protein expression of interleukin-8 in human colorectal adenocarcinomas. Int J Colorectal Dis 27: 709-714.    6. Baier PK, Eggstein S, Wolff-Vorbeck G, Baumgartner U, Hopt UT (2005) Chemokines in human colorectal carcinoma. Anticancer Res 25: 3581-3584.    7. Cui G, Yuan A, Goll R, Vonen B, Florholmen J (2009) Dynamic changes of interleukin-8 network along the colorectal adenoma-carcinoma sequence. Cancer Immunol Immunother 58: 1897-1905.    8. Tsamis D, Theodoropoulos G, Stamopoulos P, Siakavellas S, Delistathi T, et al. (2012) Systemic inflammatory response after laparoscopic and conventional colectomy for cancer: a matched case-control study. Surg Endosc 26: 1436-1443.    9. Szkaradkiewicz A, Marciniak R, Chudzicka-Strugala I, Wasilewska A, Drews M, et al. (2009) Proinflammatory cytokines and IL-10 in inflammatory bowel disease and colorectal cancer patients. Arch Immunol Ther Exp (Warsz) 57: 291-294.    10. Abdulamir AS, Hafidh RR, Mahdi LK, Al-jeboori T, Abubaker F (2009) Investigation into the controversial association of Streptococcus gallolyticus with colorectal cancer and adenoma. BMC Cancer 9: 403.    11. Gartner EM, Griffith KA, Pan Q, Brewer GJ, Henja GF, et al. (2009) A pilot trial of the anti-angiogenic copper lowering agent tetrathiomolybdate in combination with irinotecan, 5-flurouracil, and leucovorin for metastatic colorectal cancer. Invest New Drugs 27: 159-165.    12. Fedirko V, Bostick RM, Long Q, Flanders WD, McCullough ML, et al. (2010) Effects of supplemental vitamin D and calcium on oxidative DNA damage marker in normal colorectal mucosa: a randomized clinical trial. Cancer Epidemiol Biomarkers Prev 19: 280-291.    13. Hopkins MH, Flanders WD, Bostick RM (2012) Associations of circulating inflammatory biomarkers with risk factors for colorectal cancer in colorectal adenoma patients. Biomark Insights 7: 143-150.    14. Meyerhardt JA, Ancukiewicz M, Abrams TA, Schrag D, Enzinger PC, et al. (2012) Phase I study of cetuximab, irinotecan, and vandetanib (ZD6474) as therapy for patients with previously treated metastastic colorectal cancer. PLoS One 7: e38231.    15. Osterlund P, Orpana A, Elomaa I, Repo H, Joensuu H (2002) Raltitrexed treatment promotes systemic inflammatory reaction in patients with colorectal carcinoma. Br J Cancer 87: 591-599.    16. Bunger S, Haug U, Kelly FM, Klempt-Giessing K, Cartwright A, et al. (2011) Toward standardized high-throughput serum diagnostics: multiplex-protein array identifies IL-8 and VEGF as serum markers for colon cancer. J Biomol Screen 16: 1018-1026.    17. Wu FP, Sietses C, von Blomberg BM, van Leeuwen PA, Meijer S, et al. (2003) Systemic and peritoneal inflammatory response after laparoscopic or conventional colon resection in cancer patients: a prospective, randomized trial. Dis Colon Rectum 46: 147-155.    18. Reitter EM, Ay C, Kaider A, Pirker R, Zielinski C, et al. (2014) Interleukin levels and their potential association with venous thromboembolism and survival in cancer patients. Clin Exp Immunol 177: 253-260.    19. Hopkins MH, Owen J, Ahearn T, Fedirko V, Flanders WD, et al. (2011) Effects of supplemental vitamin D and calcium on biomarkers of inflammation in colorectal adenoma patients: a randomized, controlled clinical trial. Cancer Prev Res (Phila) 4: 1645-1654.  20. Wilkening S, Tavelin B, Canzian F, Enquist K, Palmqvist R, et al. (2008) Interleukin promoter polymorphisms and prognosis in colorectal cancer. Carcinogenesis 29: 1202-1206.    21. Walczak A, Przybylowska K, Dziki L, Sygut A, Chojnacki C, et al. (2012) The lL-8 and IL-13 gene polymorphisms in inflammatory bowel disease and colorectal cancer. DNA Cell Biol 31: 1431-1438.    22. Mustapha MA, Shahpudin SN, Aziz AA, Ankathil R (2012) Risk modification of colorectal cancer susceptibility by interleukin-8 -251T>A polymorphism in Malaysians. World J Gastroenterol 18: 2668-2673.    23. Landi S, Moreno V, Gioia-Patricola L, Guino E, Navarro M, et al. (2003) Association of common polymorphisms in inflammatory genes interleukin (IL)6, IL8, tumor necrosis factor alpha, NFKB1, and peroxisome proliferator-activated receptor gamma with colorectal cancer. Cancer Res 63: 3560-3566.    24. Cacev T, Radosevic S, Krizanac S, Kapitanovic S (2008) Influence of interleukin-8 and interleukin-10 on sporadic colon cancer development and progression. Carcinogenesis 29: 1572-1580.    25. Lurje G, Zhang W, Schultheis AM, Yang D, Groshen S, et al. (2008) Polymorphisms in VEGF and IL-8 predict tumor recurrence in stage III colon cancer. Ann Oncol 19: 1734-1741.    26. Bondurant KL, Lundgreen A, Herrick JS, Kadlubar S, Wolff RK, et al. (2013) Interleukin genes and associations with colon and rectal cancer risk and overall survival. Int J Cancer 132: 905-915.    27. Gunter MJ, Canzian F, Landi S, Chanock SJ, Sinha R, et al. (2006) Inflammation-related gene polymorphisms and colorectal adenoma. Cancer Epidemiol Biomarkers Prev 15: 1126-1131.    28. Zhang W, Stoehlmacher J, Park DJ, Yang D, Borchard E, et al. (2005) Gene polymorphisms of epidermal growth factor receptor and its downstream effector, interleukin-8, predict oxaliplatin efficacy in patients with advanced colorectal cancer. Clin Colorectal Cancer 5: 124-131. | The article didn’t give IL-8 related survival curve or HR.  The article evaluated the survival with CSS and DFS, without the OS data we need.  The article evaluated the survival with PFS, without the OS data we need.  The article just gave the mean level of IL-8, and we were unable to get cut-off point and assess it.  The article just gave the mean level of IL-8, and we were unable to get cut-off point and assess it.  The article just gave the mean level of IL-8, and we were unable to get cut-off point and assess it.  The article just gave the mean level of IL-8, and we were unable to get cut-off point and assess it.    The article just gave the mean level of IL-8, and we were unable to get cut-off point and assess it.  The article just gave the mean level of IL-8, and we were unable to get cut-off point and assess it.  The article just gave the mean level of IL-8, and we were unable to get cut-off point and assess it.    The article didn’t give the data we concerned.    The article didn’t give the data we concerned.    The article didn’t give the data we concerned.    The article didn’t give the data we concerned.    The article didn’t give the data we concerned.  The article and another study were based on the same population and the main authors were same.  The article didn’t give the data we need.  The article didn’t give the data we concerned.  The article didn’t give the data we concerned.  The article was for the association between IL-8 polymorphisms and CRC.  The article was for the association between IL-8 polymorphisms and CRC.  The article was for the association between IL-8 polymorphisms and CRC.  The article was for the association between IL-8 polymorphisms and CRC.  The article didn’t give the data we concerned.  The article was for the association between IL-8 polymorphisms and CRC.  The article was for the association between IL-8 polymorphisms and CRC.  The article was for the association between IL-8 polymorphisms and CRC.  The article was for the association between IL-8 polymorphisms and CRC. |
